# Public health detailing to increase expedited partner therapy for chlamydia and gonorrhea in Maryland: Changes in awareness and implementation among prescribing community providers

**DOI:** 10.1016/j.pmedr.2021.101530

**Published:** 2021-08-26

**Authors:** Rachel Milkovich, Christina Schumacher, Xueting Tao, Tina Lamidi, Ashley Edwards, Elisabeth Liebow, Kenneth Ruby, Arik V. Marcell, Jacky M. Jennings

**Affiliations:** a Center for Child and Community Health Research, Department of Pediatrics, Johns Hopkins School of Medicine, Baltimore, MD, USA; b Maryland Department of Health, Baltimore, MD, USA; c Adolescent Medicine, Department of Pediatrics, Johns Hopkins School of Medicine, Baltimore, MD, USA

**Keywords:** Public health detailing, Expedited partner therapy, Sexually transmitted illnesses, STI treatment, Chlamydia, Gonorrhea

## Abstract

The objective of this evaluation was to assess the use of public health detailing in a pilot program to increase Expedited Partner Therapy (EPT) uptake among community-based providers in two Maryland jurisdictions. Public health detailing is a method designed to raise awareness and increase implementation of evidence-based clinical practices by delivering educational content via one-on-one meetings with providers. EPT is a voluntary clinical practice of treating all sexual partners of patients diagnosed with STIs by prescribing medications without the provider first examining said sexual partners. The aim of EPT is to prevent STI reinfection and reduce further transmission. From April 2017 to March 2019, detailers visited community-based health care practice sites to conduct EPT detailing with providers. The effectiveness of this program was evaluated by comparing provider responses from pre- to post-detailing surveys, administered six months after detailing. Survey responses assessed EPT awareness and practices, barriers to implementation, and satisfaction with detailing. The proportion of providers (170) aware of EPT for treating chlamydia and gonorrhea increased from 61.7% (114) to 99.4% (169) (p-value < 0.001). The proportion who reported prescribing EPT increased from 63.2% (72) to 86.4% (146) (p-value < 0.001). Providers reporting no barriers to prescribing EPT increased from 30.6% (52) to 55.9% (95) (p-value < 0.001). Most providers were satisfied with detailing, 95.5% (164), and 95.3% (162) preferred this method to communicate about public health measures. Detailing appears to be a strategy to improve provider awareness of EPT, increase EPT implementation, and reduce barriers to prescribing EPT.

## 1 Introduction

*Chlamydia trachomatis and Neisseria gonorrhoeae* are the two most common infections reported in the U.S. (Centers for Disease Control and Prevention, 2018). In 2018, 1,758,668 chlamydia diagnoses were reported to the Centers for Disease Control and Prevention (CDC), at a rate of 539.9 cases per 100,000 persons. In the same year, 583,405 gonorrhea diagnoses were reported, at 179.1 cases per 100,000 (Centers for Disease Control and Prevention, 2018). Since 2018, chlamydia and gonorrhea cases have steadily increased among males and females, among all racial and ethnic groups (Centers for Disease Control and Prevention, 2018). Chlamydia and gonorrhea, which are often asymptomatic, pose long-term health consequences if left untreated (National Institute of Allergy and Infectious Disease, 2021). Reinfection can occur when a patient’s sexual partners are not effectively treated and increases a patient’s risk of developing reproductive complications, which occurs in 10.0–15.0% of patients in the U.S. within six months of treatment (National Institute of Allergy and Infectious Disease, 2021, Hosenfeld et al., 2009). Expedited partner therapy (EPT) is a clinical practice of treating all sexual partners of patients diagnosed with STIs by prescribing medications without the provider first examining said sexual partners (Division of STD Prevention, 2021). EPT is a voluntary practice in which an index patient may directly notify their sexual partners of their diagnosis or provide the contact information of their sexual partners for provider-assisted notification (Division of STD Prevention, 2021). EPT is not currently recommended for men who have sex with men, due to high risk of coexisting infections, including syphilis and undiagnosed HIV (Kirkcaldy et al., 2012).

The efficacy of EPT has been tested against standard referrals, in which patients are instructed to tell their sexual partners to seek care, in several randomized controlled trials. In King County, Washington, index patients receiving EPT experienced a reduction in repeat gonorrhea infections (3.0% versus 11.0% among those receiving standard partner referrals) and chlamydia infections (11.0% versus 13.0%) (Golden et al., 2005). Another study in New Orleans among male patients diagnosed with chlamydia and gonorrhea demonstrated that receipt of EPT was associated with a decrease in test positivity at one-month follow-up (23.0%), compared to those receiving standard referrals (42.7%) (Kissinger et al., 2005). A secondary analysis showed that patients given EPT were more likely to disclose to sexual partners that they had been exposed to an STI (70.6%), in comparison to standard referrals (49.1%) (Mohammed et al., 2010).

Health care providers’ awareness and implementation of EPT can promote or hinder EPT availability for patients diagnosed with chlamydia or gonorrhea. Public health detailing (“detailing”) has been shown to change and improve provider awareness and behaviors around evidence-based clinical practices (Soumerai and Avorn, 1990, Larson et al., 2006). This method draws from principles of social marketing, in which trained detailers visit health practice sites to hold individualized meetings with providers to share information and resources (Dresser et al., 2012, Ard et al., 2019). Central aspects of detailing include the provision of evidence-based tools, relationship-building with providers over time, and opening channels of communication between local health departments and providers (Dresser et al., 2012).

Detailing has been shown to be more effective than other programs that rely on distributing informational materials without in-person contact (Soumerai and Avorn, 1990, Larson et al., 2006, Dresser et al., 2012). Greiner Safi et al. (2017) found that detailing can influence behaviors among community-based providers on routine HIV screening (Greiner Safi et al., 2017). Post-detailing, 74.4% (67 of 90) of providers reported that HIV screening at their practice increased (Greiner Safi et al., 2017). Dresser et al. (2012) observed similar findings in an evaluation of detailing programs from the New York City Department of Health and Mental Hygiene (Dresser et al., 2012). Providers reported an increase in routine screenings for intimate partner violence from 14.0% to 42.0% post-detailing (p < 0.01) (Dresser et al., 2012). Dresser et al. (2012) also saw improvements in prescribing practices (Dresser et al., 2012). After receiving detailing on emergency contraception, providers increased advance prescriptions from 7.0% to 17.0% (p = 0.01) (Dresser et al., 2012). In both studies, nearly all providers were satisfied with the program and were receptive to future detailing on STI and non-STI related topics (Dresser et al., 2012, Greiner Safi et al., 2017).

Since 2015, EPT has been legally permissible in Maryland, allowing licensed providers to provide antibiotic therapy to sexual partners of patients diagnosed with chlamydia or gonorrhea (Maryland Department of Health and Mental Hygiene, 2016). Maryland law permits physicians, nurse practitioners, certified nurse midwives, physician assistants, and residents to prescribe and dispense EPT (Maryland Department of Health and Mental Hygiene, 2016). EPT is recommended for sexual partners unable or unlikely to obtain timely medical assessment, which may include those who are uninsured, lack a primary care provider, face significant barriers to accessing clinical services, or are unwilling to seek care (Maryland Department of Health and Mental Hygiene, 2016). The name and birthdate of each sexual partner is not required to prescribe or dispense EPT (Maryland Department of Health and Mental Hygiene, 2016). If these are unknown, the written designation EPT is sufficient for the pharmacist to fill the prescription (Maryland Department of Health and Mental Hygiene, 2016). Despite the legality of EPT, uptake among providers remains low (McCool-Myers et al., 2020, Kissinger, 2014). To pilot whether detailing may be an effective approach to increase EPT provision, the objectives of this analysis were to (1) evaluate the impact of detailing on EPT awareness and implementation, (2) collect information around barriers to EPT implementation, and (3) determine satisfaction with detailing among community-based providers pre- to post-detailing.

## 2 Material and methods

The evaluation of this EPT detailing program was conducted using a pre-post design and was approved by the Johns Hopkins Medicine Institutional Review Board. The detailing program was administered by two, full-time detailers over two years, from April 2017 to March 2019. Detailing was performed with providers in a convenience sample of community-based practice sites in two Maryland jurisdictions, Baltimore City and Prince George’s County.

### 2.1 Site selection

Public health surveillance data on reported cases of chlamydia and gonorrhea from January 2015 to December 2016 were utilized to select high morbidity jurisdictions, followed by practices for detailing. Two out of 23 jurisdictions were selected based on a ranked list of high morbidity counties in Maryland. These were Baltimore City and Prince George’s County, both of which had the highest morbidity rates statewide in 2016 (Maryland Department of Health and Mental Hygiene, 2018). Further, these jurisdictions had the 37th and 38th highest chlamydia rates and 30th and 60th highest gonorrhea rates, respectively, among all U.S. counties and independent cities in 2018 (Centers for Disease Control and Prevention, 2018). Within Baltimore City and Prince George’s County, community-based health care sites were eligible for detailing if they (1) were in the top 25th percentile of a ranked list of practices and/or census tracts by morbidity, and (2) self-identified as specializing in primary care, family medicine, obstetrics and gynecology, pediatrics (inclusive of adolescent populations), or internal medicine.

### 2.2 Public health detailing Intervention and evaluation

The target population for detailing were providers able to prescribe or dispense EPT, including physicians, nurse practitioners, certified nurse midwives, physician assistants, and residents.

Two detailers were hired for this pilot program. Selection characteristics for the detailers included being personable, having an interest in fieldwork, and an ability to clearly communicate health information. Detailer training focused on an “all office approach,” which recognizes that any clinic staff member can be a champion for EPT. Detailers received one week of formal training on EPT key messages, EPT regulations in Maryland, selected readings on the efficacy of EPT, and a comprehensive review of detailing materials, including the STI Action Kit and EPT script pad. The STI Action Kit is a comprehensive resource guide with information on STIs, including EPT, which was developed through informal focus group discussions with primary health and infectious disease providers, as well as with local academic experts (Greiner Safi et al., 2017, STI action kit. Johns Hopkins Medicine, 2021). An important aspect of detailer training was role-playing, in which detailers practiced engaging with clinical and non-clinical staff members. Training did not instruct detailers to conduct detailing for a specified amount of time. Rather, they were encouraged to be as efficient as possible, recognizing that a major barrier to effective detailing are time constraints for providers (Greiner Safi et al., 2017).

Based on social marketing principles, detailing sessions at practice sites began with unscheduled visits, in which detailers introduced themselves as representatives of the local health department to demonstrate credibility (Dresser et al., 2012, Ard et al., 2019). They then determined the number of providers working at each practice site and requested meetings with eligible providers. Often, this required liaising with front desk clerks, lead physicians, and clinic managers. Detailers administered face-to-face surveys with providers prior to detailing (i.e., pre-detailing survey). Survey responses were keyed directly into a Research Electronic Data Capture (REDCap) database. Once the survey was complete, detailing began, which included a review of key messages, an overview of state EPT regulations, and discussion of EPT contraindications, including intimate partner violence. Detailers also provided resources to providers, including the STI Action Kit and EPT script pads.

Maryland law on EPT does not outline specific guidance for implementation, such as patient-delivered partner therapy (PDPT) versus an EPT prescription. Informal focus groups with providers indicated that provision of PDPT was cost-prohibitive and electronic medical records (EMRs) could not be used to generate a prescription for someone other than the index patient. Detailers were prepared to respond to questions related to these concerns and provided pre-printed prescription pads for EPT-specific medication to facilitate distribution. At the conclusion of each session, detailers asked providers to commit to some level of action, ranging from reviewing the STI Action Kit with colleagues, to fully implementing EPT to all eligible patients. Detailers completed field notes after each visit, describing the site’s needs, questions, and receptiveness to detailing. As part of follow-up, detailers made additional scheduled and unscheduled visits to practice sites over a six-month period to provide additional resources, answer questions, continue relationship-building, and conduct detailing with other providers and staff members. After six months, detailers administered face-to-face surveys (i.e., post-detailing survey) with eligible providers, which included those who received a pre-detailing survey and at least one follow-up detailing visit.

### 2.3 Measures

All measures were assessed in pre- and post-detailing surveys unless specified. Surveys were administered face-to-face with eligible providers and detailers at practice sites.

#### 2.3.1 Provider characteristics

Providers were asked to specify their provider type. Eligible providers included those able to prescribe or dispense EPT: physicians, nurse practitioners, physician assistants, certified nurse midwifes, residents, or other.

#### 2.3.2 Practice characteristics

Providers were asked about patient volume in a typical week overall and for patients, ages 15–24, (the highest morbidity age range for chlamydia and gonorrhea), as well as the number of patients they diagnosed with chlamydia and gonorrhea in a typical week.

#### 2.3.3 EPT awareness (objective 1, primary outcome)

Awareness of EPT for treating gonorrhea and chlamydia was asked with single item: “Do you know that providers in Maryland can treat sex partners of patients with chlamydia or gonorrhea, also called Expedited Partner Therapy or EPT?”

#### 2.3.4 EPT implementation practices (objective 1, primary outcome)

Providers were asked whether they offered EPT for treating gonorrhea and/or chlamydia, and specifically, whether they offered EPT in accordance with each of the following Maryland guidelines on patients who should be offered EPT: (1) patients with a presumptive diagnosis of chlamydia and gonorrhea; (2) patients with laboratory confirmed chlamydia or gonorrhea diagnoses; and 3) patients unsure of their sexual partner’s STI status.

#### 2.3.5 Barriers to EPT implementation (objective 2, primary outcome)

Providers were asked to report on any barriers to EPT implementation for themselves or their practice site.

#### 2.3.6 Satisfaction with detailing (objective 3, primary outcome)

Post-detailing at the six-month visit, providers were asked about their satisfaction with detailing, including whether providers viewed detailing as a preferred method to deliver evidence-based recommendations, and as an opportunity to build relationships with local health departments.

#### 2.3.7 Other practices related to EPT (secondary outcomes)

Providers were asked whether they offered EPT to patients reporting intimate partner violence, a contraindication for EPT (Maryland Department of Health and Mental Hygiene, 2016); their clinic followed STI retesting recommendations; they were aware of CDC recommendations to retest positive gonorrhea and chlamydia patients three months after treatment; they offered verbal counseling to promote STI retesting; they reported chlamydia and gonorrhea diagnoses to the health department; and they completed a sexual history for new patients or separately, for those with a specific STI concern.

### 2.4 Statistical analyses

Summary statistics were generated to describe providers, patient volumes, and diagnoses in a typical week. Primary and secondary outcome data (except satisfaction) were summarized for the pre- and post-detailing visits and statistically tested. The McNemar test for binary matched paired data and the paired *t*-test were utilized to evaluate changes in provider responses from pre- to post-detailing. Satisfaction outcomes were assessed and summarized at post-detailing only. All analyses were conducted using Stata v 15.0 (Stata Corp, College Station, TX) and a p-value of <0.05 was considered statistically significant.

## 3 Results

Eighty eligible practice sites were identified including 12 high morbidity practices (7 in Baltimore City, 5 in Prince George’s County) and 68 practices located in high morbidity areas (56 in Baltimore City, 24 in Prince George’s County). Among these, 76.2% (61) practices agreed to participate in the detailing program. Within the 61 practices, 240 providers were approached, of whom 96.3% (231) agreed to participate. The main reason for non-participation was that practices (16) and providers (8) reported they were too busy or had no time for the program.

All providers who agreed to participate (231) completed the pre-detailing survey, received detailing on EPT and at least one STI Action Kit. One quarter (23.8%, 55) of providers were not eligible for post-detailing surveys because they had left the practice or could not be reached after three attempts to contact them. Among the remaining 176 providers, 96.6% (170) agreed to participate in the post-detailing survey and were included in the final evaluation analysis. Those included, compared to those excluded from the final analyses, were similar by provider type (data not shown).

Among the 170 providers included in the analysis, 68.1% (115) were physicians, 26.0% (44) were nurse practitioners, 2.4% (4) were physician assistants, 0.6% (1) were certified nurse midwives, 0.6% (1) were residents, and 2.4% (4) were other health care provider types. Pre-detailing, the median number of patients seen by providers, overall and among 15–24-year old’s, in a typical week was 45 (interquartile range [IQR]: 20–73) and 12 (IQR: 4–25), respectively. The median number of chlamydia and/or gonorrhea diagnoses observed by providers in a typical week pre-detailing was 0 (IQR: 0–2) with a cumulative number of 300 diagnosed in a typical week by all providers. Post-detailing, the median number of patients seen in a typical week, overall and among 15–24-year old’s, was 40 (IQR: 25–64) and 10 (IQR: 5–20), respectively. The median number of chlamydia and/or gonorrhea diagnoses by providers within a typical week post-detailing was 0 (IQR: 0–2) with a cumulative number of 167 diagnosed in a typical week by all providers.

### 3.1 Outcomes

#### 3.1.1 EPT awareness (objective 1, primary outcome).

Awareness of EPT significantly increased post-detailing, from 67.1% (114) of providers reporting being aware of EPT at pre-detailing compared to 99.4% (169) (p-value < 0.001) (Table 1).

**Table 1:** Public Health Detailing Outcomes including EPT Awareness and Implementation Practices Pre- and Six-Month Post-Detailing Among Community Health Care Providers Receiving a Public Health Detailing Intervention for EPT, Baltimore City and Prince George’s County, Maryland, April 2017 – March 2019 (N = 170).

	Pre-detailing		Post-detailing		P-Value
	N	%	N	%	
**Primary Outcomes**					
*EPT Awareness*					
Awareness of EPT for treating gonorrhea and chlamydia	114	67.1	169	99.4	< 0.001
*EPT Implementation Practices*					
Offering EPT for treating gonorrhea and/or chlamydia	72	63.2	146	86.4	< 0.001
Offering EPT in accordance with Maryland guidelines to:					
presumptive chlamydia and/or gonorrhea cases	25	14.7	58	34.1	< 0.001
laboratory confirmed positive cases	56	32.9	118	69.4	< 0.001
index patients unsure of sexual partners’ STI status	5	2.9	28	16.5	< 0.001
**Secondary Outcomes**					
Awareness of intimate partner violence as contraindication of EPT	22	12.9	33	19.4	0.140
Reporting that clinics follow STI retesting recommendation	89	53.3	110	64.7	0.009
Awareness of CDC guidelines to retest positive gonorrhea and chlamydia patients three months after treatment	113	74.3	141	82.9	0.014
Offering verbal counseling to promote STI retesting	63	37.1	77	45.3	0.100
Self-report or designee reported diagnoses of positive gonorrhea and/or chlamydia to the health department	130	77.8	150	89.3	0.002
*Completing Sexual History*					
Completing sexual history for new patients	127	74.7	146	85.9	0.005
Completing sexual history for patient with specific STI concern	148	87.1	161	94.7	0.024

#### 3.1.2 EPT implementation practices (objective 1, primary outcome).

EPT implementation practices significantly increased pre- to post detailing. Sixty-three percent (72) of providers reported offering EPT for treating gonorrhea and/or chlamydia pre-detailing, with a significant increase to 86.4% (146) of providers post-detailing (p-value < 0.001). The proportion of providers offering EPT to specific patient subgroups, according to Maryland guidelines, significantly increased pre- to post-detailing (p-value < 0.001), including offering EPT to presumptive chlamydia and/or gonorrhea positive patients, 14.7% (25) to 34.1% (58); laboratory confirmed positive cases, 32.9% (56) to 69.4% (118); and index patients who are unsure of their sexual partner’s STI status, 2.9% (5) to 16.5% (28).

#### 3.1.3 Barriers to EPT implementation (objective 2, primary outcome)

The proportion of providers reporting no barriers to prescribing EPT increased from pre- to post-detailing, 30.6% (52) to 55.9% (95) (p-value < 0.001) (Table 2). Providers’ reports of the following barriers significantly decreased from pre- to post-detailing, respectively, including not being aware of current EPT regulations, 37.6% (64) to 4.1% (7); EPT is not the clinic’s policy, 15.3% (26) to 3.5% (6); and additional EPT training is needed, 33.5% (57) to 2.4% (4). Providers’ reports of EMRs not allowing EPT prescriptions decreased, not significantly, from 11.8% (20) to 9.4% (16). The following barriers remained largely the same from pre- to post-detailing, including unknown allergies of the sexual partner(s), lack of comfort prescribing EPT, EPT is too costly to patient/insurance, EPT takes too much time, and no patients eligible for EPT.

**Table 2:** Barriers Reported by Community Health Care Providers on Implementing EPT Pre- and Six-Month Post-Detailing Among Community Health Care Providers Receiving a Public Health Detailing Intervention for EPT, Baltimore City and Prince George’s County, Maryland, April 2017 – March 2019 (N = 170).

Barriers to EPT Implementation (primary outcome)	Pre-detailing		Post-detailing		P-Value
	**N**	**%**	**N**	**%**	
No barriers reported	52	30.6	95	55.9	< 0.001
Not aware of current EPT regulations	64	37.6	7	4.1	< 0.001
EPT is not the clinic’s policy	26	15.3	6	3.5	< 0.001
Need for additional training on EPT	57	33.5	4	2.4	< 0.001
Electronic Medical Records (EMR) does not allow EPT prescriptions	20	11.8	16	9.4	0.570
Unknown allergies of sexual partner(s)	14	8.2	24	14.1	0.090
I am not comfortable prescribing EPT	11	6.5	11	6.5	1.000
EPT is too costly to patient/insurance	4	2.4	5	2.9	1.000
EPT takes too much time	5	2.9	4	2.4	1.000
No patients eligible for EPT	4	2.4	5	2.9	1.000

#### 3.1.4 Satisfaction with detailing (objective 3, primary outcome)

Six-months after the initial detailing visit, 96.5% (164) of providers reported being satisfied with the detailing program, 95.3% (162) viewed detailing as a preferred method to deliver evidence-based recommendations, and 88.2% (150) saw detailing as an opportunity to build relationships with local health departments.

#### 3.1.5 Other practices related to EPT (secondary outcomes)

Increased awareness and practices of guidelines related to chlamydia and gonorrhea treatment were observed pre- to post-detailing (Table 1). There was an increase, although not significant, in providers who recognized intimate partner violence as a contraindication to offering EPT, pre- to post-detailing 12.9% (22) to 19.4% (33) (p-value = 0.14). The proportion of providers reporting that their clinic follows STI retesting recommendations increased, from 53.3% (89) to 64.7% (110) (p-value = 0.009). The proportion of providers aware of the CDC recommendation to retest positive gonorrhea and chlamydia patients three months after treatment increased significantly, from 74.3% (113) to 82.9% (141) (p-value = 0.014). The proportion of providers who reported offering verbal counseling to promote STI retesting three months after treatment increased, although not significantly, from 37.1% (63) to 45.3% (77) (p-value = 0.10). Providers indicating that they reported a positive gonorrhea and chlamydia diagnosis to the health department through self-report or a designee significantly increased, from 77.8% (130) to 89.3% (150) (p-value = 0.002). Post-detailing, providers significantly reported more frequently completing a sexual history for a new patient visit, 74.7% (127) to 85.9% (146) (p-value = 0.005), or for those with a specific STI concern, 87.1% (148) to 94.7% (161) (p-value = 0.024).

## 4 Discussion

This evaluation suggests that detailing may effectively deliver evidence-based clinical practice information on state and national EPT regulations to community-based providers. Our results demonstrate that detailing can improve providers’ EPT awareness and implementation with sustained awareness and change over a six-month period. Additionally, providers were satisfied with the detailing experience. After receiving detailing for EPT, providers were more familiar with EPT guidelines and increased their offering of EPT to patients. Ninety-seven percent of providers reported that they were satisfied with detailing. The majority viewed detailing as a preferred method of delivering evidence-based practices and as an opportunity to build relationships with the local health department. These findings have important implications for increasing provision of EPT among community-based providers and ultimately, for decreasing onward and repeat STI infections. These findings contribute to a greater literature on detailing work in other health care areas, suggesting that detailing is a tool to successfully deliver information on public health practices to community-based providers.

Based on our results, we found several areas for improvement. Adverse events related to EPT are rare, including allergic reactions among sexual partners (Stekler et al., 2005). However, post-detailing, more providers expressed concern about prescribing EPT when partner’s allergies were unknown. This corroborates previous work that concerns about adverse reactions to EPT were commonly reported by providers and pharmacists. Future work to increase the uptake of EPT should address concerns about potential allergic reactions to medications and associated liability. The inability to print EPT prescriptions from EMRs remains as an important barrier. While we did not track this data formally, we found that pre-printed paper prescription pad refills were frequently requested by providers and may have helped overcome this barrier. EMRs should be modified to allow for EPT prescriptions to be printed, but we found that the paper prescription pads offered an interim solution for implementation. The focus of this detailing program was on increasing provider awareness and practices related to EPT. However, offering EPT is part of a multi-step process and includes other stakeholders (i.e., index patient, sexual partner(s), and pharmacists). Future work could expand detailing to pharmacists, to ensure awareness and prevent EPT prescriptions from being rejected.

Several limitations should be considered when interpreting results. All outcomes were self-reported measures collected through face-to-face surveys between detailers and providers, which may be subject to social desirability bias. This could lead to an overestimation of providers offering EPT. About one-quarter of providers interviewed pre-detailing were not eligible for the post-detailing survey, and therefore, were not included in analyses. Although, those who were included versus excluded did not differ by provider type. This evaluation design did not allow for the measurement of chlamydia and gonorrhea reinfection rates pre- and post-detailing; future work should consider incorporating this. Among three primary outcomes for objective one, one outcome measured whether EPT awareness increased following the detailing program and may have been inadvertently leading. If leading, this question would have likely decreased the difference between a pre- and post-detailing evaluation. Yet, this outcome was significantly different between the two time points. In addition, the positive significant findings of this outcome are concomitant, with the positive findings of the other primary outcomes for this objective, suggesting a similar and positive pattern of results. Future work might consider comparing self-reports in EPT knowledge and behaviors among providers to actual delivery of EPT to patients and/or their sex partners. Additionally, detailing programs may consider strengthening messaging to providers on contraindications, such as intimate partner violence.

## 5 Conclusion

This evaluation suggests that detailing to community-based providers located in high morbidity areas, or practicing in high morbidity patient settings, is an effective method to increase provider awareness of and implementation practices for EPT. Additional research is needed to further quantify and address barriers and facilitators to EPT implementation, such as the ability to generate EPT prescriptions from EMRs, to address provider concerns of unknown allergies, and to assess cost-effectiveness of detailing. Detailing appears to be a key strategy to improve providers’ awareness of EPT, increase EPT implementation, and reduce barriers to prescribing EPT.

## Funding

This evaluation was supported by the Centers for Disease Control and Prevention STD-Prevention and Control for Health Departments, Maryland Department of Health and Mental Hygiene, and the National Association of County and City Health Officials.

## CRediT authorship contribution statement

**Rachel Milkovich:** Writing – original draft. **Christina Schumacher:** Formal analysis. **Xueting Tao:** Formal analysis, Data curation. **Tina Lamidi:** Investigation, Project administration. **Ashley Edwards:** Investigation. **Elisabeth Liebow:** Conceptualization, Funding acquisition. **Kenneth Ruby:** Conceptualization, Funding acquisition. **Arik V. Marcell:** Writing - review & editing. **Jacky M. Jennings:** Conceptualization, Methodology, Supervision, Funding acquisition, Writing - review & editing.

## Declaration of Competing Interest

The authors declare that they have no known competing financial interests or personal relationships that could have appeared to influence the work reported in this paper.
